# In silico investigation of the puzzling dopamine effects on excitability and synaptic plasticity in hippocampal CA1 pyramidal neurons

**DOI:** 10.1038/s41598-025-17694-8

**Published:** 2025-09-25

**Authors:** Enrico Manara, Andrea Mele, Michele Migliore

**Affiliations:** 1 Institute of Biophysics, National Research Council, Palermo, Italy; 2 Centro di Ricerca in Neurobiologia-D. Bovet, Department of Biology and Biotechnologies-C. Darwin, Sapienza University, 00185 Rome, Italy

**Keywords:** CA1, Dopamine, STDP, K_A_, Computational model, Computational biophysics, Computational neuroscience

## Abstract

It has been shown that in the CA1 region of the hippocampus, dopamine modulates memory functions by influencing spike-timing-dependent plasticity (STDP) and intrinsic neuronal properties. Although experimental findings have suggested potential mechanisms, their detailed interplay remains incompletely understood. Here, using a realistic CA1 pyramidal neuron model, we have investigated the possible effects of dopaminergic modulation on a neuron’s signal integration and synaptic plasticity processes. The results suggest a physiological plausible explanation for the puzzling experimental observation that long-term potentiation (LTP) increases in spite of a reduction in the neuron’s excitability, and explains why physiological dopamine levels are necessary for LTP induction. The model suggests experimentally testable predictions on which ion channel kinetic properties can modulate the interplay between synaptic plasticity and neuronal excitability, thereby identifying potential molecular targets for therapeutic intervention.

**Supplementary Information:**

The online version contains supplementary material available at 10.1038/s41598-025-17694-8.

## Introduction

Dopamine (DA) is a neuromodulator that acts on multiple different receptors^1^. Like other neuromodulators, it exerts its effects by interacting with G-protein coupled receptors, which in turn modify the molecular structure of channels after biochemical signaling cascade. These modifications can alter the channel’s voltage response^2^ or lead to the insertion of new transmembrane protein^3^. In the CA1 region, DA is synthesized and released by the ventral tegmental area (VTA) and locus coeruleus^4^. It modulates Spike-Timing Dependent Plasticity (STDP) through multiple mechanisms^5–8^ and broadly influences plasticity in the whole hippocampus^3,9–11^. In rodent CA1, DA has been demonstrated to play a pivotal role in the formation of aversive memories^12,13^, the morphine-induced conditioned place preference^14^, the optimal functioning of place cells^15,16^ and novelty-facilitated spatial LTP^17^.

Experimental findings^6^ have shown the complex role of DA in rats CA1 hippocampal slices, where it exhibited seemingly contradictory effects. Physiological levels of DA were found to reduce neuronal excitability, but were also necessary for the induction of early LTP. In contrast, slices prepared with a sucrose-based solution containing 48% less DA exhibited higher firing frequencies, but failed to show increased plasticity at the Schaffer collateral synapses when stimulated with an STDP protocol . This phenomenon was reproduced across various Δt intervals and with stronger STDP-inducing paradigms. The application of 20 µM DA to the recording solution restored both synaptic plasticity and excitability to the levels observed under higher DA conditions. Interestingly, synaptic plasticity was dependent on NMDA receptors and was inhibited by a D1 receptor antagonist.

To study in more detail the interplay among the different cellular processes involved in these effects, computational models can be very helpful. For striatal medium spiny neuron (MSN), computational models have been developed to simulate DA modulation of intrinsic neuronal conductances and synaptic currents^18–21^. Moyer et al.^20^ suggested that D1 receptor activation reduces SK current, thereby leading to bistability in MSNs. Lindroos et al.^19^ employed an experimentally constrained model to study DA modulation under both in vitro- and in vivo-like conditions. In the latter, they focused on the timing dependency between action potentials and DA-activated substrates. Their findings indicate that a reduction in fast K_A_ current (via KV4.2 channels) is required to increase neuronal excitability. Several CA1 models simulate DA effects by detailing its molecular cascade, addressing how LTP/LTD synaptic specificity is maintained despite the diffusion of intracellular signaling molecules. The aim of these models was to elucidate the interaction between various effectors to explain how high frequency stimulation (HFS) or low frequency stimulation (LFS) lead to LTP/LTD induction and maintenance, see, e.g., Refs.^22–25^. Schmalz and Kumar^26^ developed a single-compartment biophysical model based on the Hodgkin-Huxley formalism, examining LTP and LTD induction at Schaffer collateral CA3-CA1 synapses through HFS/LFS. Their model integrates findings from multiple experiments to provide a unified understanding of how the D1/D5 agonists concentration and the relative timing between HFS/LFS and DA influence synaptic plasticity. Izhikevich^27^ proposed a minimal spiking network model based on cortical architecture, simulating DA STDP modulation to take into consideration the rewarding component. In this model, an eligibility trace generated by nearly coincident firing leads to synaptic strengthening when a reinforcement signal, represented by DA input, arrives 1–3 s later. However, despite their contributions, these models are unable to explain why a lower-than-normal DA level can prevent LTP induction in spite of making the neuron more excitable^6^.

To address this gap, we developed a morphologically and biophysically detailed CA1 pyramidal neuron model that accounts for experimental findings and yields experimentally testable predictions regarding the mechanisms underlying DA-dependent effects. Our results suggest that DA can exert its effects on synaptic plasticity by reducing the activation of dendritic K_A_ channels (thereby enhancing backpropagating action potentials (bAP)), and on neuronal excitability through an increase of the BK conductance. The model allows to make experimentally testable predictions on which ion channel properties could restore synaptic plasticity and neuronal excitability, offering insights into potential molecular targets for therapeutic interventions under conditions characterized by pathologically low DA activity.

## Methods

All simulations were carried out using the NEURON simulation environment (NEURON v8.2^28^;). All model and simulation files will be available for download and use on the ModelDB (https://modeldb.science/) and EBRAINS-Italy (https://www.ebrains-italy.eu/resources/models) databases. Experimental data not explicitly reported in Edelmann and Lessman^6^ were extracted using an online extraction tool^29^.

**Fig. 1:**
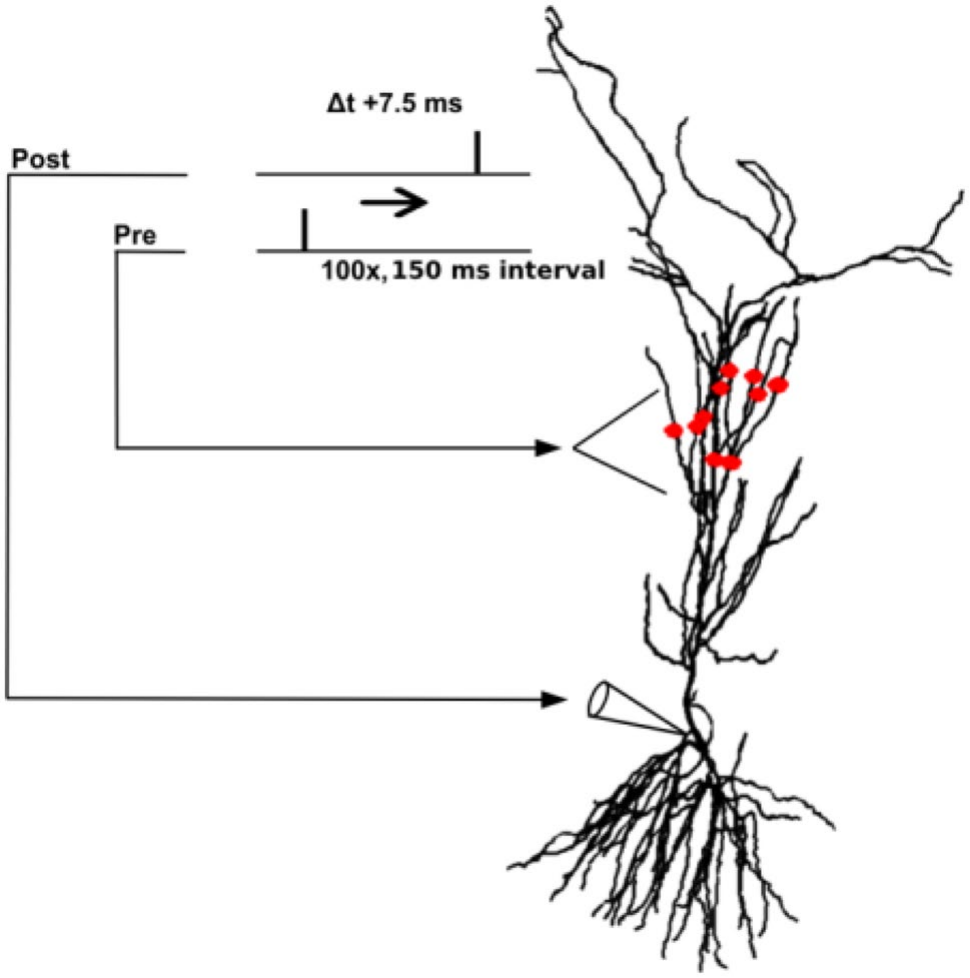
The CA1 pyramidal neuron morphology and STDP protocol used in this work. The red markers indicate typical synaptic dendritic locations used to study LTP induction.

It should be stressed that, in implementing a model for a specific set of experimental findings, we usually concentrate our attention to find an overall configuration of parameters reproducing those features that represent the scientific problem at study, rather than fitting scattered individual parameters that would require many additional experimental constraints or information that are not available. In this particular work, we focused on reproducing, at the same time, LTP induction properties and the number of spikes as a function of the input current under control and low Dopamine conditions. We started from the model by Miceli et al.^30^, (ModelDB a.n. 148,094), which employed a CA1 pyramidal neuron morphology from an adult Sprague–Dawley rat (ID 9068802a) (Fig. 1). Consistent with experimental observations, the resting membrane potential was set to − 70 mV, with a membrane time constant of ≈28 ms, in line with multiple experimental reports (e.g. Ref.^31–33^). The model included several types of active currents: a sodium current (I_Na_), a delayed rectifier (I_KDR_); a distal and proximal K_A_ current (I_KA_); a nonselective, hyperpolarization-activated current (I_h_), two types of calcium channels (a low-threshold T-type current, I_CaT_, and a long-lasting L-type current, I_CaL_), along with a simplified calcium extrusion mechanism. BK and SK channels, responsible for the fast (fI-AHP) and slow (sI-AHP) Ca^2+^-dependent potassium currents, respectively, were also included over the entire somato-dendritic tree. In the dendrites, I_KA_ and I_h_ channels were expressed with a linear increase of the peak conductance with distance from the soma, and a M-type K^+^ current (I_KM_) current was added to the soma and the axon^34^. Ion-channel peak conductances were manually calibrated by trial and error until the model’s I/O behavior was in very good qualitative agreement with that of Edelmann and Lessmann^6^, and the final peak conductance values are listed in Table 1.

**Table 1:** Current type and relative maximal conductance (S/cm^2^) over cell compartment as a function of somatic distance (x; µm).

Current	Compartment	Values
I_Na_	Soma, Axon Apical and Basal Dendrites	0.215 0.043
I_DR_	Soma, Axon Apical and Basal Dendrites	0.125 0.025
I_KA_prox_ I_KA_prox_ (< 100 µm from soma)	Soma, Axon Apical and Basal Dendrites	0.069 0.069 * (1 + distance/100)
I_KA_dist_ (> 100 µm from soma)	Apical and Basal Dendrites	0.069 * (1 + distance/100)
I_KM_	Soma Axon	2e-5 1e-4
I_h_	Soma Apical and Basal Dendrites	1.1e-6 1.1e-6 * (1 + 3*distance/100)
I_CaT_	Soma, Apical and Basal Dendrites	4e-5
I_CaL_	Soma, Apical and Basal Dendrites	2e-3

		Normal DA	Lower DA
SK channels	Soma Apical and Basal Dendrites	1.5e-3 3e-4	5e-3 1e-3
BK channels	Soma, Apical and Basal Dendrites	1.6e-4	2.1e-5

Uniform passive properties: R_m_ 11.667 kΩ/cm^2^, C_m_ 2.4 μF/cm^2^, τ_m_ 28 ms, Temperature 35 °C

It should be stressed that individual CA1 pyramidal neurons exhibit substantial degeneracy^35^ and large experimental variability across experimental conditions (e.g. animal strain, age, recording conditions, etc.). For these reasons, many different parameter combinations could have resulted in a reasonable reproduction of the experimental findings. However, a detailed exploration of the parameter space was beyond the scope of the present study.

Synapses consisted of colocalized AMPA and NMDA components. The AMPA current was implemented with double exponential functions with raise and decay time of 0.5 and 3 ms, respectively, whereas the NMDA receptors were implemented as described in Cutsuridis et al.^36^. In any given LTP simulation, 10 synapses randomly distributed on the oblique dendrites in the *stratum radiatum* (a typical dendritic distribution is shown in Fig. 1) were activated following the experimental protocol. The average LTP measured at the soma was calculated from 50 repetitions after randomly redistributing the synapses.

STDP rules were adapted from Bianchi et al.^37^, in such a way to be consistent with the experimental findings used as a reference in this work^38^ (i.e. 100% LTP for a Δt up to 10 ms, and no change for larger Δt). The spike threshold was set at − 25 mV, so that any dendritic potential exceeding this value would indicate the occurrence of a spike. This is in agreement with experimental findings suggesting that the membrane voltage does not need to attain highly depolarized values for plasticity to occur^39^. The model uses an instantaneous, empirical weight update at each pre-post pairing, abstracting away the complex and much slower biochemical cascades to maintain computational efficiency. This is an almost universally used modeling approach when the model is also intended to be plugged in large scale networks, where a biochemically detailed implementation would require prohibitively long computational times.

## Results

**Fig. 2:**
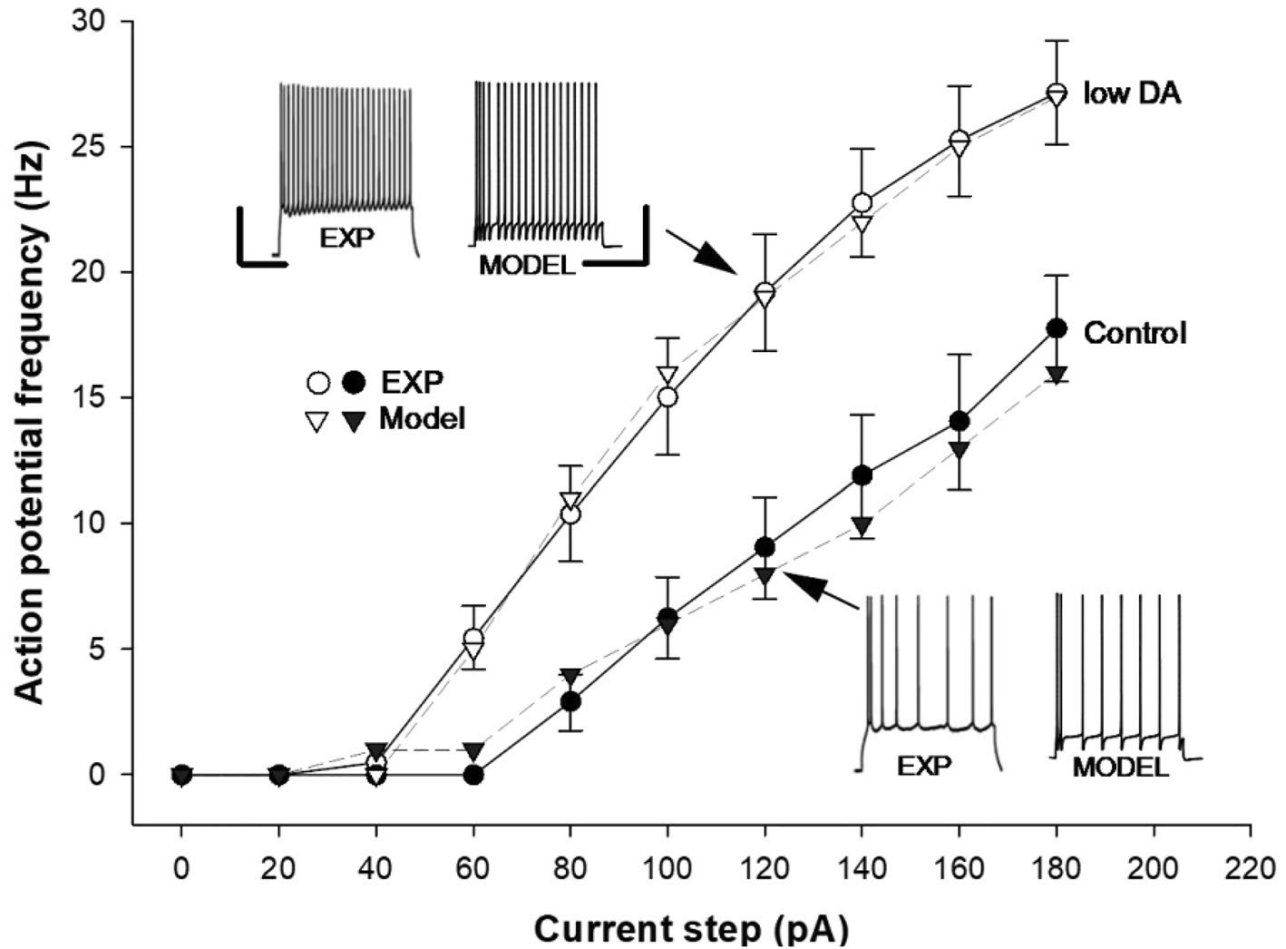
Comparison of model results with experiments (from Edelman and Lessman^6^), during 1 s long somatic current injections. A lower DA condition induces an increase in the neuron excitability. Error bars indicate ± SEM. Insets show typical somatic traces from experiments and model at current steps indicated by the arrows. Scale bars: EXP (20 mV, 500 ms), MOD (40 mV, 500 ms).

We first modeled the experimental findings under control conditions (Fig. 2, white circles) by manually adjusting the peak channels’ conductance in such a way to qualitatively reproduce the mean firing frequency as a function of somatic current injection (Fig. 2 white triangles). The final peak conductance values for all channels are reported in Table 1. Starting from this configuration, we model the experimentally observed effects of DA reduction (Fig. 2, black circles). Experimental evidence suggests that a reduced DA level can alter the *K*_*A*_ kinetics as well as Ca^2+^-dependent SK and BK conductances^40–48^. For this purpose, we directly implemented experimental findings^49^ indicating that the K_A_ inactivation time constant increased by 30% and that both activation and inactivation curves should shift negatively relative to control condition. We found that the best results were obtained with a − 5.5 mV shift (Table 2).

**Table 2:** Different distal I_KA_ kinetics and steady-state curves under normal and lower DA level conditions.

Normal DA level		Lower DA level	
Distal I_KA_		Distal I_KA_	
V_1/2_ Activation:	+ 4.5 mV	V_1/2_ Activation:	− 1 mV
V_1/2_ Inactivation:	− 50 mV	V_1/2_ Inactivation:	− 56 mV
Inactivation		Inactivation	
Time constant:	1.9 ms/10 mV	Time constant:	2.6 ms/10 mV

We also found that a BK decrease by ~ 85% and a SK increase by a factor of ~ 3 were sufficient to reproduce the experimental findings under current clamp (Fig. 2, black triangles). It should be noted that we were interested in modeling relative differences in DA levels—lower versus higher—rather than absolute “normal” versus “low” values. Consequently, our methodology prioritized capturing the biologically consistent directionality of the change induced by DA, as demonstrated across different studies^6,49^, over replicating the exact parameter values from any given experimental preparation**.** Although to the best of our knowledge, no study has directly quantified the change in BK channel conductance under dopaminergic modulation, many papers report a reduction in BK‐mediated currents (Refs.^43,47,48^). Thus, the ~ 85% downregulation of BK conductance in our model should be viewed as a specific quantitative prediction required to reproduce the Edelmann and Lessmann^6^ data. As in the experiments, low DA condition resulted in increased neuronal excitability, characterized by a lower rheobase and up to a 60% increase in firing frequency as a function of the current injection. Overall, the model successfully reproduced the trends observed in the input/output curves under both conditions in response to 1-s long somatic current injections. The experimental finding that low DA prevents LTP induction is puzzling, given that increased neuronal excitability would normally be expected to promote, rather than inhibit, synaptic plasticity. We will show in the following paragraphs how this phenomenon could be explained using the model.

To test for LTP induction, we replicated the experimental stimulation protocols for long-term synaptic plasticity^6^. Ten synapses were randomly distributed along the oblique dendrites between 150 and 400 µm from the soma, corresponding to the *stratum radiatum* layer. Each synapse contained both AMPA and NMDA components in a 1:1 ratio. A presynaptic pulse was delivered simultaneously to all synapses and paired with a single action potential (AP) elicited in the soma by a 3 ms, 1.2 nA current injection. This pairing was repeated 100 times with 150 ms long intervals, while the synapses’ locations were randomly redistributed between runs to reflect the experimental variability in synaptic activation during conditioning. The somatic AP was elicited with a + 7.5 ms delay relative to the EPSP onset, corresponding to the delay at which the largest DA-dependent differences were observed experimentally. Results using other Δt values are shown in Supplementary Fig. 1. The synaptic weight was adjusted to ensure that the membrane potential remained subthreshold (i.e., without triggering somatic or dendritic APs) throughout the conditioning protocol.

**Fig. 3:**
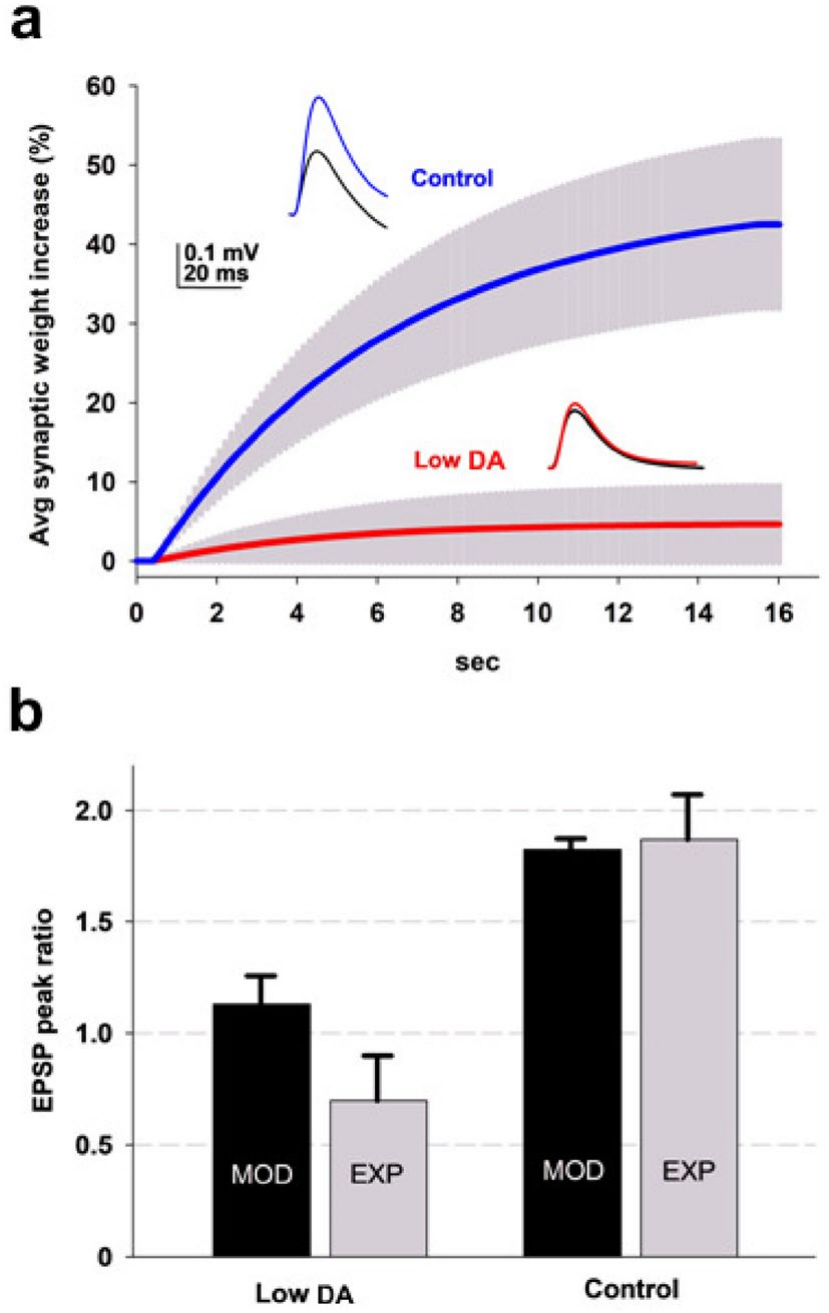
Spike-timing-dependent plasticity under control or low DA conditions. (**a**) Time course of the average synaptic weight during the conditioning protocol; the gray areas represent standard deviation; the insets show typical somatic voltage traces in response to test EPSPs before (black) and after (red and blue) the conditioning protocol. (**b**) Model results (MOD) compared with experiments (EXP) for the average peak EPSP ratio measured at the soma; note that experimental data^6^ did not reveal a statistically significant LTP under low DA conditions. Model error bars indicate ± Std. Dev., experimental error bars indicate ± SEM.

During the LTP induction protocol each synaptic weight evolved according to local dendritic active and passive properties. As shown in Fig. 3, this resulted in an average increase of approximately 40%. In contrast, under low DA conditions, the average increase was negligible. The inset in Fig. 3 shows the average somatic membrane potential traces in response to a test EPSP before and after conditioning; these traces reflect the overall depolarization envelope from individual synaptic locations propagated to the soma. Their average increase is represented in Fig. 3b compared with experimental findings. Under both normal and low DA levels, the model was in quantitative agreement with experiments, with only a 13% increase under low DA compared to an 83% increase under control conditions. It may be argued that some LTD was induced under low DA in the experiments (grey bar for Low DA in Fig. 3). However, in the original paper^6^ clearly noted that the plasticity measured after pairing protocol was not significantly different from the negative-control condition (presynaptic stimulation alone; see their Result section). The relatively large experimental variability was not considered here. The model was thus able to reproduce the puzzling experimental findings suggesting that lowering the DA level will prevent LTP in spite of an increase in the neuron’s excitability.

**Fig. 4:**
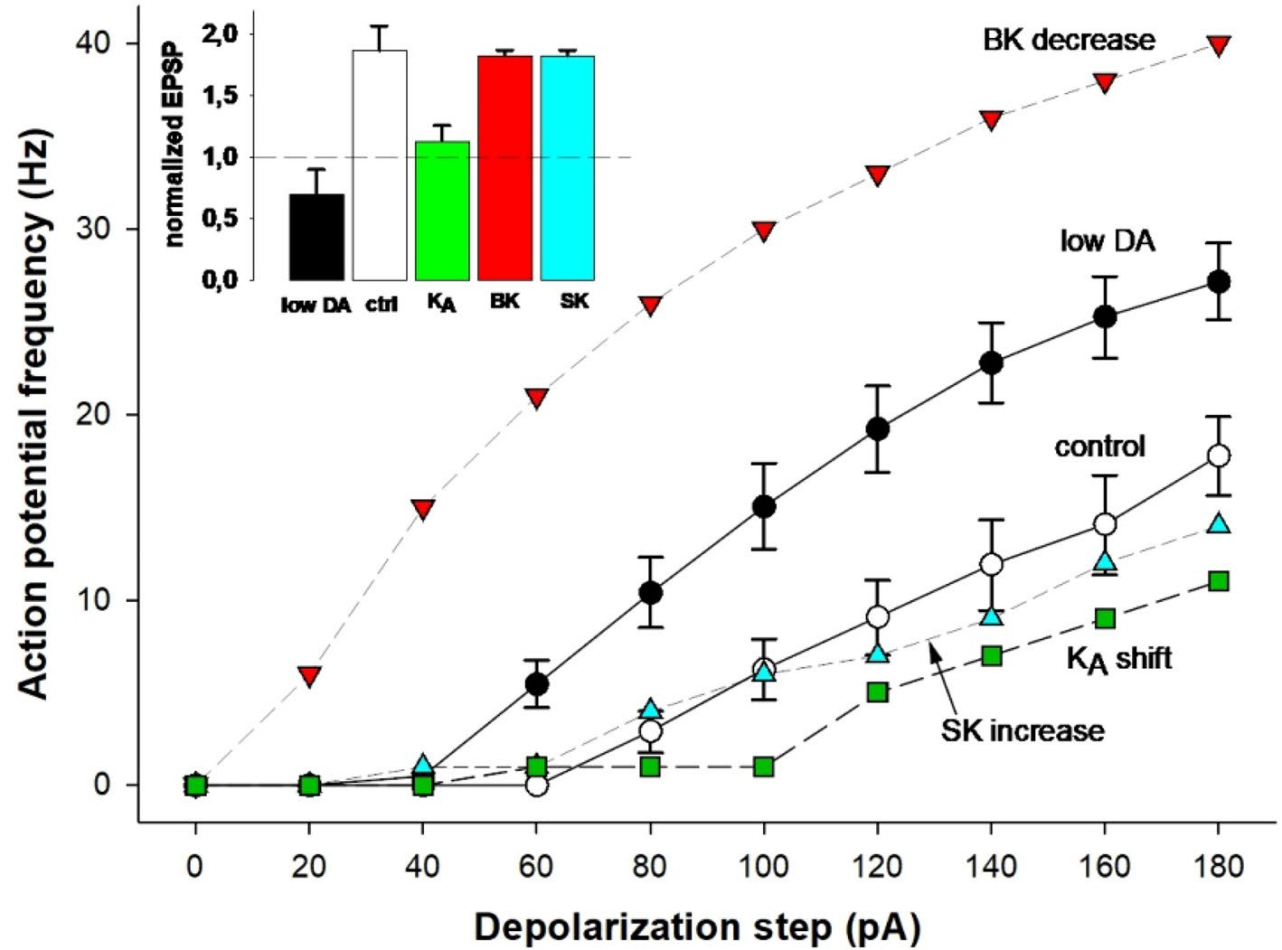
Neuron’s excitability after DA-dependent modulation of individual ionic conductance. The different plot shows the average firing frequency as a function of the somatic current step under control (white symbols) and low DA conditions (black symbols), compared with the results obtained by applying low DA conditions to individual ionic current components. Inset shows normalized EPSP. It demonstrates that changing only the K_A_ shift under low-DA condition is both necessary and sufficient to abolish tLTP, whereas BK and SK modulations have negligible effects on synaptic plasticity.

The overall effect of low DA on the neuron’s firing properties is caused by the interplay among the different ionic currents, which exert opposing effects: whereas the (negative) shift of dendritic K_A_ kinetics decreases excitability by increasing its dampening effect on action potentials backpropagation, the BK decrease will increase excitability by reducing the overall amount of transmembrane K^+^ current. We investigate this issue in more detail by highlighting the individual effect of low DA on each of the involved currents. In Fig. 4, the input/output (I/O) curves under normal (white markers) and low DA conditions (black markers) are compared. We used the results under normal conditions as a reference, then one parameter at a time was switched to its corresponding value under low DA conditions. By changing only the dendritic K_A_ kinetics (Fig. 4, green) or only the SK conductance (Fig. 4, cyan) the neuron became even less excitable, with respect to control condition (Fig. 4, white). In contrast, reducing the BK current alone (red markers) produced a significant increase in excitability, exceeding what was observed under low DA conditions (Fig. 4, black). It should be noted that other forms of dopaminergic modulation can contribute to reducing the excitability. For instance, in vitro experiments in the entorhinal cortex have shown that DA increases I_h_ conductance^50^. In principle, this upregulation could offset the excitability increase resulting from reduced K_A_ current. To test this, we ran a series of simulations in which we progressively increased peak I_h_ conductance in lieu of elevated AHP currents in the control condition (see Supplementary Fig. 2). None of these manipulations resulted in a good agreement with the experimental input–output curve^6^. Moreover, since in pyramidal CA1 neurons an I_h_ increase cannot improve dendritic bAP, it failed to rescue LTP induction (data not shown). These results suggest that the effect of DA on BK may have a major role in modulating the intrinsic electrophysiological properties of CA1 pyramidal neurons. As shown in the inset of Fig. 4 and discussed in the next section, only the K_A_ shift under low-DA condition is both necessary and sufficient to abolish tLTP, whereas BK and SK modulations have negligible effects on synaptic plasticity. These results suggest that the specific effect on dendritic K_A_ kinetics is the mechanism responsible for the lack of LTP induction under low DA conditions.

**Fig. 5:**
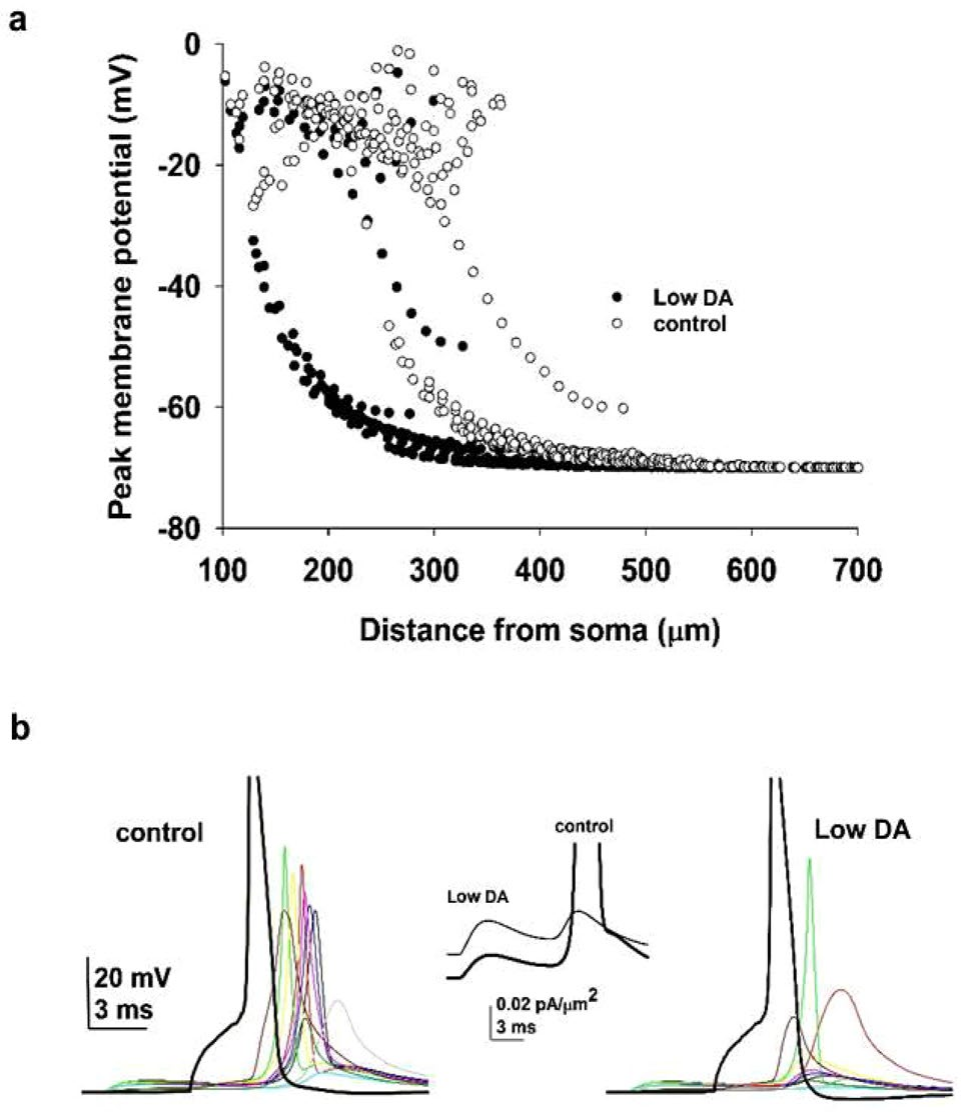
Low DA conditions hinder AP backpropagation. (**a**) Peak membrane potential in apical dendrite segments as a function of the distance from the soma, in response to a single AP elicited by a short somatic current pulse (1 nA, 2 ms), under control (white) or low DA (black) conditions. (**b**) Membrane potential at the soma (black) and at synaptic input dendritic locations (colored traces) during pairing of a synaptic input with a somatically generated AP with a 7.5 ms delay under control (left plot) and low DA (right plot) conditions. Note that the AP does not propagate in the dendrites under low DA conditions. The somatic trace is truncated. The middle plot shows the K_A_ current under the two conditions.

Synaptic plasticity induced by a STDP protocol crucially depends on the association between a synaptic input and bAP. Under control conditions, K_A_ kinetics in the dendrites are similar to those in the soma and proximal dendrites^49^. Although their density increases with distance from soma, a bAP is still able to invade at full amplitude all oblique dendrites in the *stratum radiatum*. We demonstrate this effect in Fig. 5a (white circles), where we plot the peak membrane potential reached in all membrane compartments during a bAP. The peak depolarization was more than − 60 mV up to 400 µm from the soma, allowing the association between synaptic inputs and bAPs necessary for LTP induction. Under low DA conditions, the negative shift in the V_1/2_ activation of the dendritic K_A_ current amplifies the K_A_-dependent dampening effect of K_A_ on bAPs^51,52^. This is evident in Fig. 5a (black circles) for dendrites in the *stratum radiatum* (approximately 200–500 µm from the soma). Note that in the more distal dendrites in the *stratum lacunosum moleculare*, the bAP is largely unaffected by DA, in agreement with the experimental suggestion that DA does not alter synaptic integration in the perforant pathway^53^. Between 50 and 400 µm from the soma, only 12% of membrane segments exceeded the − 25 mV STDP threshold under low DA conditions, compared to 59.1% in control. The differential back-propagation of action potentials in the oblique dendrites can thus be the mechanism responsible for the lack of LTP induction under low DA conditions. This can be appreciated by considering the typical traces shown in Fig. 5b, where we plot somatic and dendritic membrane potential during pairing of a bAP with an EPSPs on all synapses. This resulted in a large local depolarization under control conditions (Fig. 5b, left), which was proven to be sufficient to induce LTP (see Fig. 3), in contrast with what happened under low DA conditions (Fig. 5b, right), where the local depolarization was hindered by a stronger K_A_ current, as demonstrated by the typical local K_A_ current traces plotted in the middle panel of Fig. 5b.

**Fig. 6:**
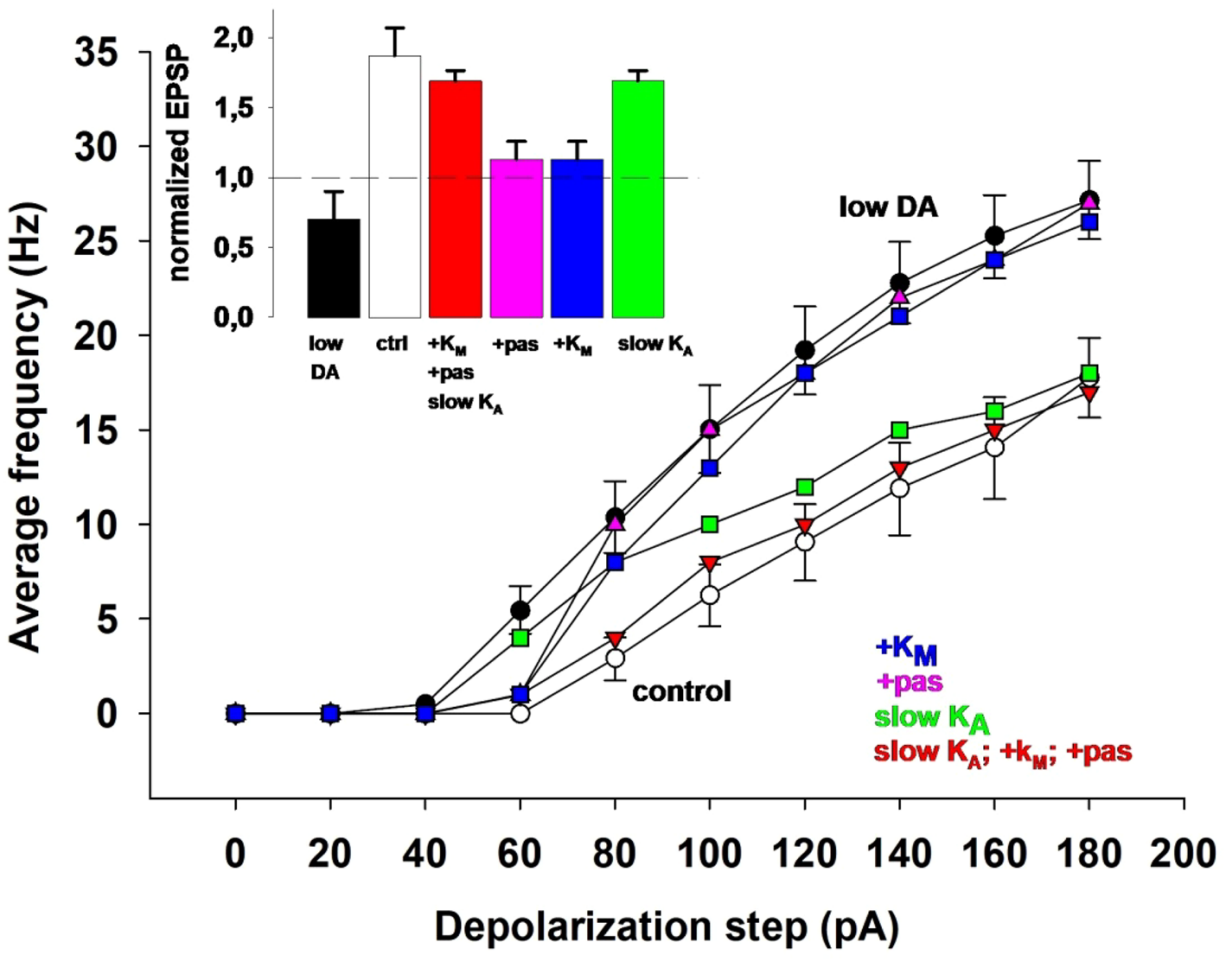
Rescuing control conditions under low DA. The plot shows firing frequency as a function of input current for control, low DA, and various single- or combined-current manipulations, serving as an alternative to the previous I_KA_ and I_AHP_ currents adjustments used to mimic normal DA levels. The inset displays normalized EPSPs. While individual current modulations alone fail to restore LTP and neuronal excitability, combining them successfully rescues these properties. Model error bars indicate ± Std. Dev., experimental error bars indicate ± SEM.

The absence of LTP under low DA conditions can thus be attributed to the specific DA-mediated changes on the K_A_ activation. We next asked whether a compensatory change or a pharmacological intervention modulating membrane and/or ion channel kinetic properties (unaffected by DA) could rescue control levels of LTP and cell’s excitability. As we have seen in the previous paragraphs, the lack of LTP induction under low DA conditions is caused by the shift in the K_A_ activation curve, which prevents bAPs propagation into the oblique dendrites. It may be argued that a simple reduction of K_A_ peak conductance could thus, in principle, restore LTP by allowing bAPs to invade dendrites. However, this would not be a suitable modulation, since it would increase even more the neuron’s excitability. Instead, we hypothesized that a slower activation time constant could simultaneously reduce excitability and support LTP. The rationale for this hypothesis is that a slower K_A_ activation would still hinder an isolated (fast) bAP; instead, the relatively slower depolarizing envelope of an EPSP would be able to deactivate the current, allowing for a better synaptic integration of a bAP with an EPSP. The model supported this hypothesis, as shown in Fig. 6: LTP was rescued to its control level (see inset in Fig. 6, green bar), and the neuron’s excitability was reduced, although not in the entire range of stimulation currents (Fig. 6, compare green and white circles). Therefore the model predicts that a modulatory change in the *K*_*A*_ alone would not be enough to restore control conditions under low DA; some additional membrane property should also be considered. Among those not affected by DA are the passive properties and the K_M_. From this point of view, an increase in the membrane conductance would reduce excitability by increasing the cell input resistance, whereas an increase in K_M_ would reduce somato-axonic excitability without affecting synaptic integration, as K_M_ is not expressed in the apical dendrites of CA1 pyramidal neurons^34^. We found that individual changes in the membrane conductance (+ 17% increase, Fig. 6 pink symbols) or a threefold K_M_ increase (Fig. 6, blue symbols) were insufficient to significantly reduce the cell’s excitability or rescue LTP induction (Fig. 6, pink and blue bars). However, when combined together with a slower K_A_, they not only support LTP induction (Fig. 6, red bars) but also enable the neuron to exhibit excitability similar to control levels across the entire range of tested currents (Fig. 6, red symbols), despite low DA condition.

Interestingly, all three targets—*K*_*A*_, K_M_-current, and leak conductances—can be pharmacologically modulated, thus supporting the possibility of pharmacological intervention to restore LTP induction and normal excitability under low dopamine. For example, phrixotoxins, among other effects, can slow the activation kinetics of Kv4 (*K*_*A*_) channels^54^. SCR 2682 upregulates Kv7 (M-current) channel expression and conductance^55^. Finally, passive K⁺ currents can be augmented by arachidonic acid, which activates TREK-1 channels^56^, or by alkalinizing the extracellular solution to potentiate TASK-1 channels^57^, thereby reducing intrinsic neuronal excitability.

## Discussion

The aim of this study was to investigate, using a computational model, the potential mechanism(s) that could explain the puzzling experimental findings suggesting that a reduction in the DA signaling will block LTP induction while increasing a cell’s excitability. The model suggested that a low DA level exhibits these seemingly contradictory effects via an up- and down-regulation of the K_A_ and BK Potassium currents, respectively. With these two mechanisms in place, the model successfully reproduced experimental data (Figs. 2,3). The model suggests that the negative shift of the K_A_ activation curve (similar to what experimentally observed between the proximal and distal dendrites^51^ increases this current, thereby reducing excitability and the possibility for a bAP to invade oblique dendrites; although the concurrent downregulation of BK current makes the neuron overall more excitable, it still cannot prevent the reduction in bAP dendritic invasion. The net effect is a poor local summation, which prevents LTP induction despite an increase in somatic spiking.

For Ca^2+^-dependent channels, the literature presents somewhat conflicting data regarding their DA modulation in the hippocampus, usually measured through their effects on the membrane after-hyperpolarization properties (I_AHP_). In general, the BK and SK modulation appears to be distinct, as neurons may exhibit one, both, or neither of them. Some studies suggest DA-dependent increase^43,45,48^, while other findings suggest a reduction, either involving the activation of beta-adrenergic receptors^46^ or via DA receptors themselves^44^. Additionally, there is evidence that D1 receptors increase IAHP while D2 receptors decrease it^48^. Pockett^43^ observed an increased SK conductance in rat hippocampal slices. Moreover, bath-applied DA reduced the afterdepolarization (ADP) through a negative shift in the spike repolarization point. This effect was ascribed to an increased BK conductance. In other brain regions, such as the striatum and prefrontal cortex (PFC), DA appears to have mixed effects on I_AHP_^58–61^.

Regarding K_A_ modulation, experimental evidence suggests that DA can reduce the *K*_*A*_ current in various brain regions^62,63^. Observations in the stomatogastric ganglion of the spiny lobster indicate that DA exerts opposing effects on K_A_, depending on the specific class of neuron being examined^64^. Yang and Dani^65^ observed that, in the synapse between the medial perforant pathway and the dentate gyrus, a D1/D5 agonist reduced K_A_ current, increased the magnitude of tLTP, widened its temporal window, and enabled tLTP even with negative Δt. This phenomenon may be attributed to the amplification of bAPs and have implications for object recognition^66^.

Our model also suggests that DA exerts differential effects on various channels, reflecting its observed opposing effects on the input/output (I/O) curve^67–69^. In CA1, D1 activation can lead to either a reduction^70^ or an increase^71,72^ in intrinsic excitability. This occurs despite the well-established molecular pathways of D1-like and D2-like receptors, which respectively activate and inhibit PKA/PKC^1^. As a result, both increases and decreases in firing frequency may arise from the downregulation or upregulation of the same ion channels. One possible explanation is the differential binding of DA to D1-like or D2-like receptors, depending on phasic versus tonic type of release, given their distinct neurotransmitter affinities^73,74^. Additional factors such as the presence of heteromeric receptors^75^, co-release with GABA or glutamate^76^, DA volume transmission^74^, the influence on local interneuron^53^ and receptor-independent mechanisms^77^ add further complexity to this neuromodulatory system.

Concerning synaptic plasticity, the effect modeled in this study represents just one of the many potential consequences of DA on synaptic plasticity. It has been postulated that DA availability is a prerequisite for STDP in the hippocampus, although the precise pre- and post-synaptic firing patterns necessary for its induction remain unclear due to the varying levels of DA reported across studies (e.g., Ref^78^). Nevertheless, the impact of DA on STDP appears to be complex. For example, in cultured hippocampal neurons, where endogenous DA is absent, STDP can be still induced. Adding DA to the bath engages D1 receptors, extending the effective time window for LTP, even with negative Δt, and reducing the threshold of pairing stimuli required for LTP^5^. These seemingly divergent findings highlight that dopamine’s gating of STDP depends critically on experimental preparation. In dissociated hippocampal cultures^5^, chronic absence of dopamine likely induces recruiting of alternative gating factors such as BDNF/TrkB which could preserve LTP in the absence of endogenous DA. Conversely, in acute slices^6^, endogenous DA levels are reduced but not absent, remaining below the threshold required for plasticity and thus not invoking homeostatic compensation. Additionally, while GABA_A receptors are blocked in slices with picrotoxin, GABA_B receptors remain functional and may further constrain STDP window expansion, a factor absent in cell culture where the inhibitory network is not present. Another study also reported a lowered pairing threshold for LTD induction, with implications for spatial memory and novelty detection^79^. It has been shown that the combined action of D1 and D2 receptors can as well retroactively convert LTD into LTP when DA is applied immediately after the conditioning protocol^8^. DA is also necessary for the induction of LTP, even with a limited number of stimulus repetitions, via the preferential action of D2 receptors^7^, while D1 receptors increase LTP magnitude in CA1^80,81^ and inhibit depotentiation^82^. The molecular substrates modulated by dopamine are numerous and we did not attempt to simulate all of them in detail and at the same time. For example, the dopamine-dependent mechanisms that allow a widening of the window for LTP^5^ could depend on a greater persistence over time in calcium elevation following EPSP/bAP, or on a lowering of the threshold required for LTP. However, in the context of the experimental findings that we used as a reference to implement our model, this effect could not explain the difference in the intrinsic excitability.

In the present study, we thus propose that K_A_ and BK modulation may represent the main mechanisms through which DA facilitates LTP induction while reducing neuronal excitability. One possible limitation of this study is that the model simulates a static DA condition, whereas in vivo DA concentrations can change relatively rapidly. However, this aspect was beyond the scope of the present work, which was based on in vitro experimental data. Identifying the specific substrates responsible for these effects could help inform pharmacological approaches to counteract the impact of substance abuse^14^ or to treat conditions associated with DA dysfunction, such as Parkinson’s disease^83^. The model can be straightforwardly adapted to study further alternative modulations, such as a I_Na_ modulation^70,84,85^, or a decrease in I_CaT_^86^, to determine which best reproduces the available data.

## Supplementary Information

Supplementary Information.

## Data Availability

All model and simulation files will be available for download and reuse at the ModeldDB (https://modeldb.science/) and EBRAINS-italy (https://www.ebrains-italy.eu/resources/models) depositories.
