# Supplementary material for: In silico investigation of the puzzling dopamine effects on excitability and synaptic plasticity in hippocampal CA1 pyramidal neurons
Source: Sci Rep. 2025 Sep 25;15:32822. doi: 10.1038/s41598-025-17694-8 (PMC12464202; doi:10.1038/s41598-025-17694-8)
Supplement: Supplementary file 1 — Supplementary Information. [file 41598_2025_17694_MOESM1_ESM.pdf]

In silico investigation of the puzzling Dopamine effects on excitability and synaptic plasticity in hippocampal CA1 pyramidal neurons

Enrico Manara[1], Andrea Mele[2], and Michele Migliore[1,*]

[1]Institute of Biophysics, National Research Council, Palermo, Italy
[2]Centro di Ricerca in Neurobiologia-D.Bovet, Department of Biology and Biotechnologies-C.Darwin, Sapienza University, Rome, I-00185, Italy
[*]michele.migliore@cnr.it

Supplementary figures

Supplementary figure 1

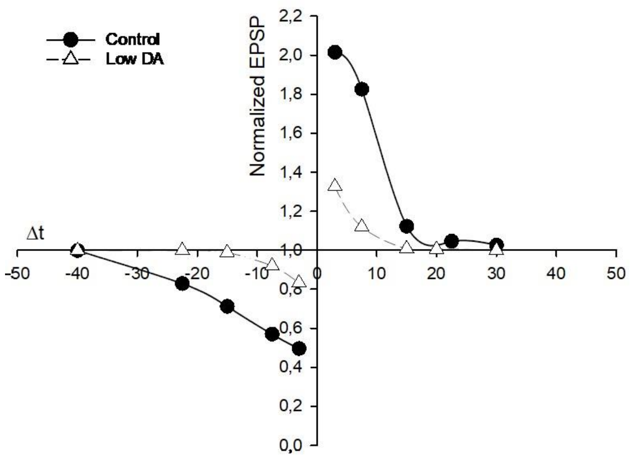

Supplementary figure 1. Timing-dependent synaptic plasticity under control and low DA conditions. Normalized EPSP amplitude is plotted as a function of the pre- to post-synaptic spike interval (Δt), calculated as described in the main simulations. Positive Δt leads to LTP, whereas negative Δt results in LTD. Nevertheless, in the presence of low DA, both phenomena are significantly reduced or abolished. Each data point corresponds to a single run.

Supplementary figure 2

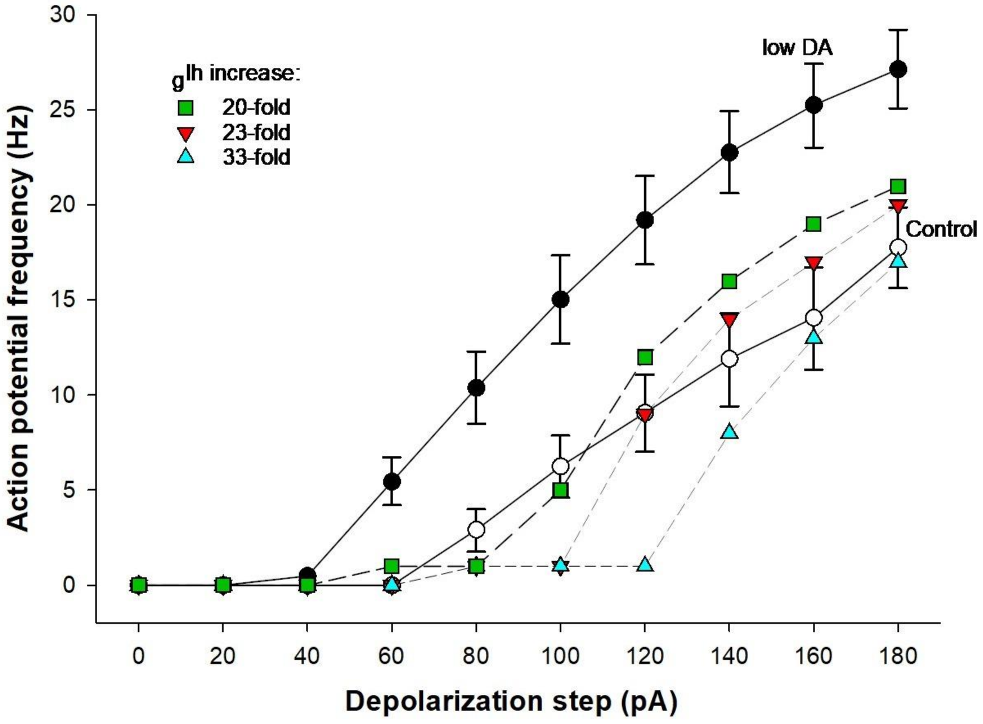

Supplementary figure 2. I_h Upregulation as an alternative to AHP Current Modulation. The same stimulation protocol previously employed was used to generate I/O curves. Under control conditions, with DA modulation of K_A current, progressively increasing I_h conductance failed to replicate the precise I/O relationship experimentally reported. The upregulation was applied proportionally to all neuronal segments, with higher absolute conductance occurring in apical dendrites.
